# Supplementary material for: Causal Relationship between Aging and Anorexia Nervosa: A White-Matter-Microstructure-Mediated Mendelian Randomization Analysis
Source: Biomedicines. 2024 Aug 16;12(8):1874. doi: 10.3390/biomedicines12081874 (PMC11351342; doi:10.3390/biomedicines12081874)
Supplement: Supplementary file 1 [file biomedicines-12-01874-s001.zip › Supplementary Figure/Figure S4/Singe SNP plot of MR analysis for TL to ubm-b-2048.pdf]

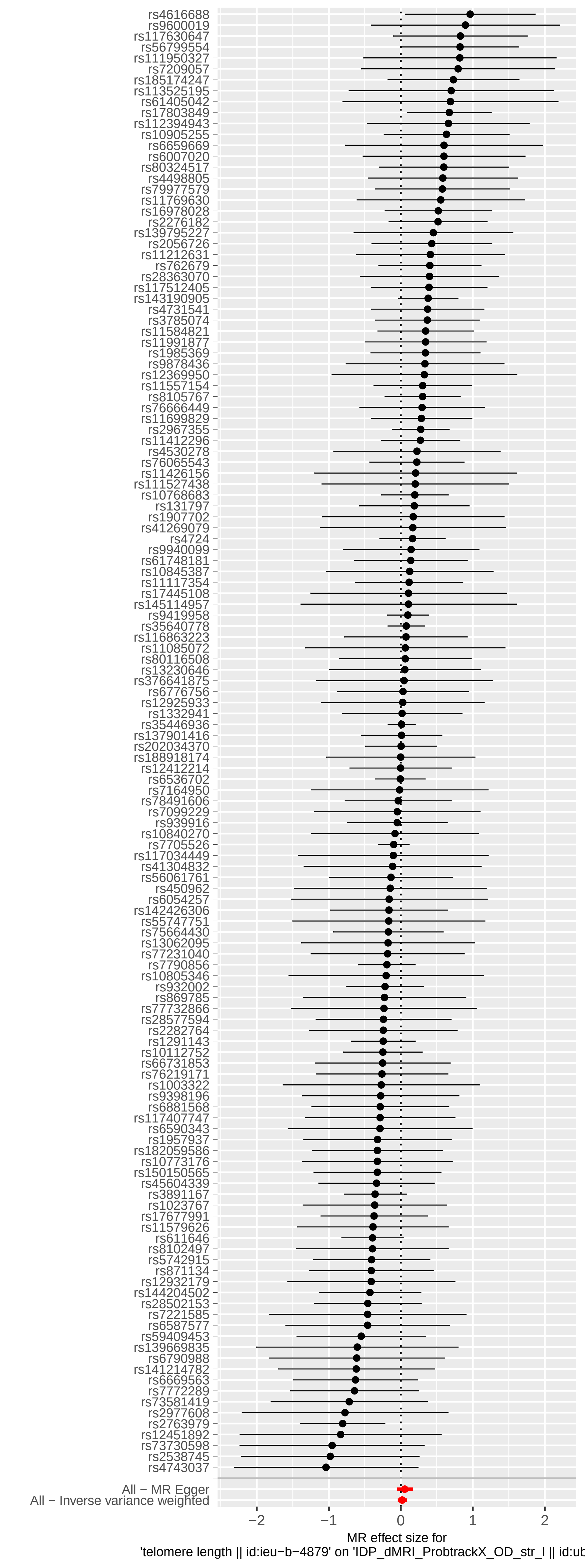
rs4616688
rs9600019
rs117630647
rs56799554
rs111950327
rs7209057
rs185174247
rs113525195
rs61405042
rs17803849
rs112394943
rs10905255
rs6659669
rs6007020
rs80324517
rs4498805
rs79977579
rs11769630
rs16978028
rs2276182
rs139795227
rs2056726
rs11212631
rs762679
rs28363070
rs117512405
rs143190905
rs4731541
rs3785074
rs11584821
rs11991877
rs1985369
rs9878436
rs12369950
rs11557154
rs8105767
rs76666449
rs11699829
rs2967355
rs11412296
rs4530278
rs76065543
rs11426156
rs111527438
rs10768683
rs131797
rs1907702
rs41269079
rs4724
rs9940099
rs61748181
rs10845387
rs11117354
rs17445108
rs145114957
rs9419958
rs35640778
rs116863223
rs11085072
rs80116508
rs13230646
rs376641875
rs6776756
rs12925933
rs1332941
rs35446936
rs137901416
rs202034370
rs188918174
rs12412214
rs6536702
rs7164950
rs78491606
rs7099229
rs939916
rs10840270
rs7705526
rs117034449
rs41304832
rs56061761
rs450962
rs6054257
rs142426306
rs55747751
rs75664430
rs13062095
rs77231040
rs7790856
rs10805346
rs932002
rs869785
rs77732866
rs28577594
rs2282764
rs1291143
rs10112752
rs66731853
rs76219171
rs1003322
rs9398196
rs6881568
rs117407747
rs6590343
rs1957937
rs182059586
rs10773176
rs150150565
rs45604339
rs3891167
rs1023767
rs17677991
rs11579626
rs611646
rs8102497
rs5742915
rs871134
rs12932179
rs144204502
rs28502153
rs7221585
rs6587577
rs59409453
rs139669835
rs6790988
rs141214782
rs6669563
rs7772289
rs73581419
rs2977608
rs2763979
rs12451892
rs73730598
rs2538745
rs4743037
All − MR Egger
All − Inverse variance weighted
−2
−1
0
1
2
MR effect size for
'telomere length || id:ieu−b−4879' on 'IDP_dMRI_ProbtrackX_OD_str_l || id:ub
